# Structured Communication: Teaching Delivery of Difficult News with Simulated Resuscitations in an Emergency Medicine Clerkship

**DOI:** 10.5811/westjem.2015.1.24147

**Published:** 2015-03-12

**Authors:** Sangeeta Lamba, Roxanne Nagurka, Michael Offin, Sandra R. Scott

**Affiliations:** Rutgers University, New Jersey Medical School, Department of Emergency Medicine, Newark, New Jersey

## Abstract

**Introduction:**

The objective is to describe the implementation and outcomes of a structured communication module used to supplement case-based simulated resuscitation training in an emergency medicine (EM) clerkship.

**Methods:**

We supplemented two case-based simulated resuscitation scenarios (cardiac arrest and blunt trauma) with role-play in order to teach medical students how to deliver news of death and poor prognosis to family of the critically ill or injured simulated patient. Quantitative outcomes were assessed with pre and post-clerkship surveys. Secondarily, students completed a written self-reflection (things that went well and why; things that did not go well and why) to further explore learner experiences with communication around resuscitation. Qualitative analysis identified themes from written self-reflections.

**Results:**

A total of 120 medical students completed the pre and post-clerkship surveys. Majority of respondents reported that they had witnessed or role-played the delivery of difficult news, but only few had real-life experience of delivering news of death (20/120, 17%) and poor prognosis (34/120, 29%). This communication module led to statistically significant increased scores for comfort, confidence, and knowledge with communicating difficult news of death and poor prognosis. Pre-post scores increased for those agreeing with statements (somewhat/very much) for delivery of news of poor prognosis: comfort 69% to 81%, confidence 66% to 81% and knowledge 76% to 90% as well as for statements regarding delivery of news of death: comfort 52% to 68%, confidence 57% to 76% and knowledge 76% to 90%. Respondents report that patient resuscitations (simulated and/or real) generated a variety of strong emotional responses such as anxiety, stress, grief and feelings of loss and failure.

**Conclusion:**

A structured communication module supplements simulated resuscitation training in an EM clerkship and leads to a self-reported increase in knowledge, comfort, and competence in communicating difficult news of death and poor prognosis to family. Educators may need to seek ways to address the strong emotions generated in learners with real and simulated patient resuscitations.

## INTRODUCTION

Optimal care for the critically ill or injured patient includes caring, compassionate communication and support for the family during and after patient resuscitation.^1^ In the emergency department (ED), resuscitation events often end in patient death or uncertain and poor outcomes.^2^–^5^ Traditionally, for both graduate and undergraduate trainees in emergency medicine (EM), the main focus is on acquiring the *technical* resuscitation skills, as exemplified by the Advanced Cardiac Life Support (ACLS) and Advanced Trauma Life Support (ATLS) courses.^6^–^9^ Little, if any, attention is directed towards teaching trainees how to deliver news of uncertain prognosis or patient death after the resuscitation efforts.^8^–^10^ Trainees are also rarely debriefed after an intense, emotional resuscitation or taught the skills of self-reflection as a means of addressing their emotions or coping with patient death. As a result, many trainees may feel unprepared to deal with the stress and emotions that may result from these highly charged resuscitation encounters.^8^,^10^ To address this gap, the EM resuscitation curriculum would benefit from the addition of opportunities for trainees to practice delivering difficult news and to provide support for the family.^11^–^14^

Simulation is increasingly used to teach critical skills, including communication, since it provides a safe and realistic environment for active learning by allowing for trial and error.^15^–^18^ Appropriate feedback to learners in the simulated setting may help shape future trainee to patient/family conversations.^18^–^22^ The principles of adult learning and Kolb’s experiential learning theories also support the use of simulation in education.

Our project is designed to teach skills of effective communication in high-stress situations that surround ED resuscitations. We use a hybrid simulation curriculum where case-based simulated resuscitation scenarios are supplemented with a structured communication that immediately follows the technical training. Our overall goal is to teach medical students in the EM clerkship how to compassionately communicate with families of critically ill and dying patients. We describe here the curriculum implementation. The main outcomes are pre-post self-assessments by learners to rate their knowledge, competence, and comfort with delivering difficult news to family. The secondary aims of our project are to 1) encourage students to use written reflective inquiry for communication skills development and 2) analyze these written learner experiences with the goal to inform future case scenario and curriculum design.

## METHODS

### Study Setting and Target Population

The educational project was conducted in a large academic, urban, tertiary care teaching hospital with approximately 100,000 ED patient visits per year. The study period was from July 2011 through May 2012. The target population consisted of fourth-year medical students on a four-week mandatory EM clerkship. The faculty facilitators were EM board certified and one facilitator was also certified in Hospice and Palliative Medicine. The institutional review board approved the study. The curriculum design and implementation was supported by the Y.C. Ho/Helen and Michael Chiang Foundation administered and guided by the expert faculty at the Harvard Medical School Center for Palliative Care.

### Description of ‘communication and self-reflection’ learning activities

**Figure 1:**
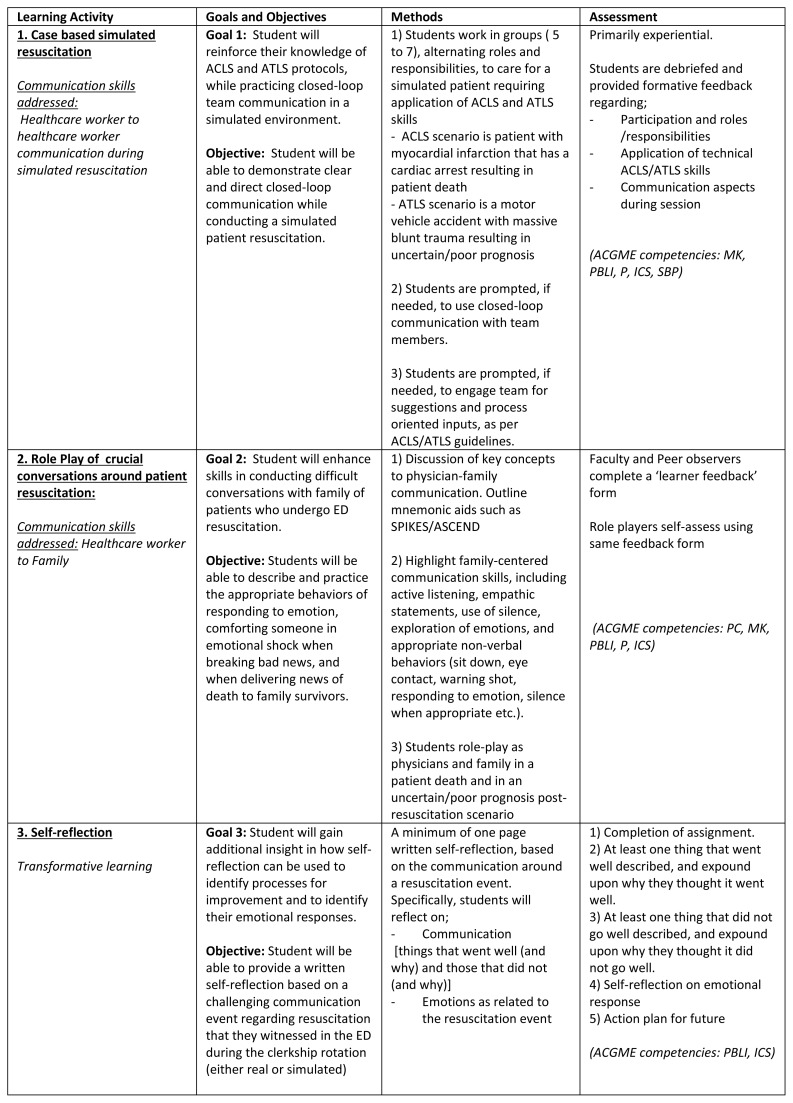
Communication and self-reflection educational module related learning activities. *ACLS,* advanced cardiac life support; *ATLS,* advanced trauma life support; *ACGME,* accreditation council for graduate medical education; *MK*, medical knowledge; *PBLI,* practice-based learning and improvement; *P,* professionalism; *ICS,* interpersonal and communication skills; *SBP*, systems based practice; *ED,* emergency department; *SPIKES*, setting, patient perception, invitation, emotions/empathy, strategy/summary; *ASCEND,* anticipation, summary, concerns elicited, exploration/explanation, next steps, documentation; *PC*, patient care

The curriculum is grounded in Kolb’s experiential theory in which the learner ‘touches all the bases’ of thinking, experiencing, acting and reflecting (Figure 1). Concrete experiences activate prior knowledge; observations are translated into concepts; the student can then actively test new concepts; this enables experiences and reflections that in turn promulgate the cycle.^23^

The communication and self-reflection educational module supplements pre-existing technical resuscitation training in the clerkship. The technical component consisted of two case-based simulated resuscitation scenarios (one addresses ACLS and the other ATLS skills). Trainees practice on a high-fidelity human adult simulation mannequin. Both simulation case scenarios with objectives as well as step-by-step outlines and competencies are available via MedEd portal.^16^ The communication skills addressed included reinforcement of closed-loop communication during simulated resuscitation. The simulated resuscitation training (ATLS and ACLS skills) was followed by the students role-playing as physician and family members to deliver news of uncertain or poor prognosis and/or death of simulated patient. Students took turns to play the role of the physician delivering difficult news and then received feedback from faculty and peer observers. Figure 1 outlines the overall goals, objectives, methods, assessment and Accreditation Council on Graduate Medical education (ACGME) competencies addressed with the learning activities for the communication module. The students were also asked to provide a written self-reflection based on a challenging communication event regarding resuscitation that they witnessed in the ED setting (real) or in the simulated encounter. They were asked to describe at least one thing that went well and one thing that did not go well. Students reflected on their emotional responses and considered an action plan to address a similar challenging situation in the future. Detailed descriptions of each learning activity, outlines and scripts for the role-play activity as well as tools for faculty development and student self and peer assessment/feedback and an outline of the self-reflection exercise are also available via the MedEd portal.^16^

### Survey Development and Content

We developed a pre- and post-clerkship survey. Pre-clerkship questions asked the student about their experiences in prior clinical rotations with delivering difficult news of death and/or poor prognosis (whether they had witnessed, role-played, or performed communication skill). In addition, the pre-clerkship survey asked students to rate their baseline knowledge, comfort, and confidence with communicating difficult news of death and poor prognosis on a scale of 1–4 (1=not at all to 4=very much).

The post-clerkship survey asked learners to rate their knowledge, comfort, and confidence regarding delivery of difficult news after completion of the simulation and communication role-play learning activity.

Since our secondary aims were to encourage reflective inquiry and to explore learner needs/experiences via written self-reflection we also added questions to address this in the pre-post surveys. We asked for the pre-EM clerkship medical student experiences with written self-reflection and its perceived value. The students were then also asked their perception of value of a written self-reflection after completion of the exercise.

The questions were piloted for readability, clarity, and content with a small group of the EM faculty as well as medical students. The comments from the pilot were reviewed and final survey was determined by author consensus.

### Survey Administration

Anonymous pre-clerkship surveys were administered to all senior medical students during orientation on the first day of the EM clerkship as a paper and pencil survey. Students placed the completed pre-clerkship survey (with no student identifiers) into an envelope, sealed it, and wrote a self-assigned, easy to remember number on the envelope. Post-clerkship surveys were similarly administered on the last day of rotation. The students identified and opened their sealed envelope and returned the stapled completed pre- and post-clerkship surveys together. We randomly assigned the returned surveys a study number and entered corresponding data into an Excel spreadsheet. Every third survey was audited (RN) to ensure accuracy of data entry. No research incentives were provided to participants.

### Data Analysis

Descriptive statistics, such as percentages, means, and medians, are provided below. We compared pre- post-clerkship dichotomous data using McNemar chi-square tests. We analyzed comparisons of pre- and post-clerkship Likert like scale ratings with Wilcoxon signed-ranks tests. P-values for comparisons of means and medians were obtained via paired-samples t-tests.

We performed qualitative analyses on experiences reported by students via the written reflections.^24^ Based on a review of 10 written self-reflections, three authors identified categories that included: “things that went well and why;” ” things that did not go well and why;” and the student’s emotional responses. We reviewed 110 written self-reflections and extracted content into categories. Subsequently recurring themes were identified within categories. Any discrepancies or conflicts were resolved by author consensus.^25^,^26^

## RESULTS

Out of the 160 senior medical students completing their fourth-year at our institution, 120 answered both the pre- and post-clerkship surveys on their EM clerkship rotations (Table 1). Eighteen students in the June rotation were not offered the survey as data collection began in July. Twenty-two students completed the pre-survey only and this data is comparable to those we included in our analyses. Students reported using e-mail as the most common means to communicate to others “what went well” or “what did not go well” with a patient encounter (76/120, 64% and 73/120, 61% respectively).

Few students (17/120, 15%) reported receiving specific training on closed-loop communication. A vast majority recalled witnessing and/or role-playing communication of difficult news of death and poor prognosis. However, few students reported personally delivering difficult news of death (20/120, 17%) and poor prognosis (34/120, 28%).

**Figure 2:**
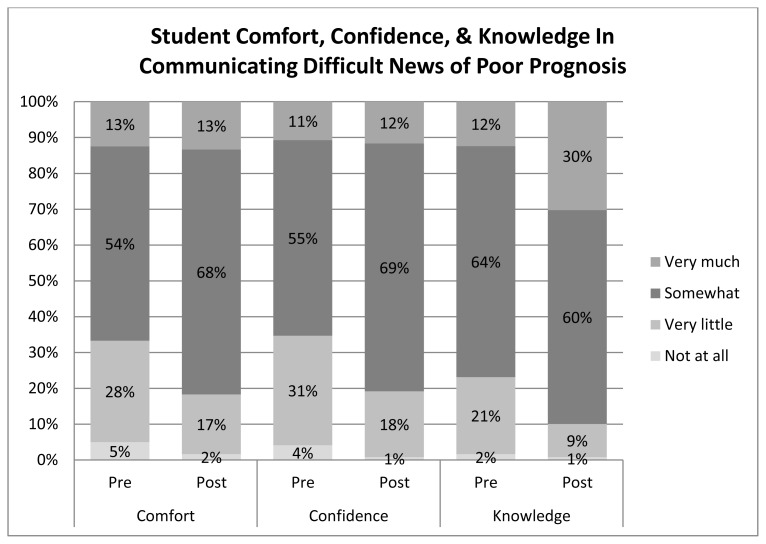
Emergency Medicine Clerkship students’ self-reported pre and post clerkship responses regarding breaking bad news of poor prognosis.

**Figure 3:**
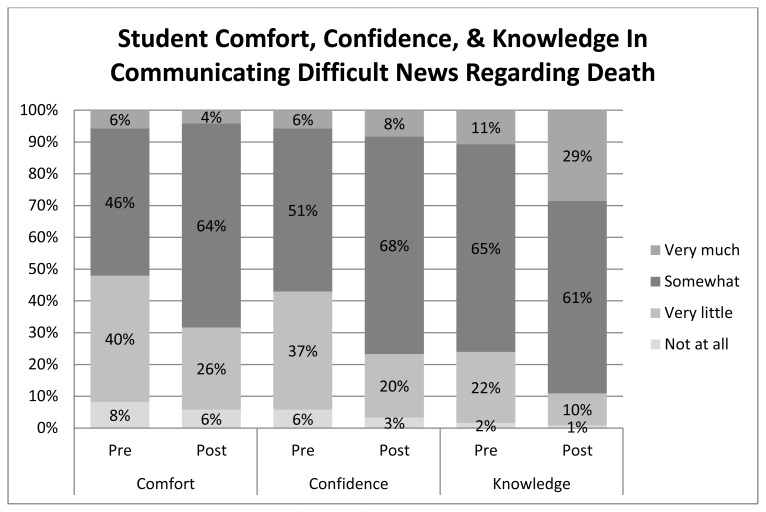
Emergency Medicine Clerkship students’ self-reported pre and post clerkship responses regarding breaking bad news of death.

After completion of the simulation and communication role-play learning activity, there was a statistically significant increase in scores related to comfort, confidence, and knowledge regarding communicating difficult news of poor prognosis and patient death (Figures 2 and 3). The largest increase was seen in the knowledge scores (Figures 2 and 3).

Of the 119 student respondents, a majority (111/119, 93%) felt that the clinical ED faculty served as either somewhat or very positive role models for communicating difficult news (Likert like scale of 1–4 with mean score of 3.26).

Prior to starting the EM clerkship an overwhelming majority of respondents (100/120, 90%) stated that they had not received specific training on written self-reflection. Similarly, a majority of students (108/120, 91%) perceived self-reflection to be a valuable tool for personal growth. Comparisons of pre- and post-clerkship survey responses revealed a statistically significant decrease in the perceived value of written self-reflection (Table 2).

### Learner experience-related themes from written self-reflections

Of the 110 written self-reflections analyzed, we explored themes expressed for real and/or simulated resuscitations under three main categories: “things that went well and why;” “things that did not go well and why;” and finally any student emotional responses identified. In general, among “things that went well,” students had positive comments about the simulation and described ED resuscitations as good role models to learn team organization and efficiency; “ED codes run as a well-oiled machine;” “ED team knew their roles well;” and “Everyone worked well together.” Students also listed that the observed ED clinician to family communication interactions were positive experiences; “Took time to explain, prompt effort to contact family.” However, among “things that did not go well,” the chaotic resuscitation setting, negative emotions, and some of the negative healthcare worker to healthcare worker communication interactions during resuscitations were cited.

Students listed strong emotional responses to both real and/or simulated resuscitation encounters:

Anxiety and stress around the overwhelming nature of resuscitation; “….the day when the responsibility will fall on me;” Feelings of a personal sense of loss, grief and of own mortality; “I am that age…that could have been me”

Sense of failure when patient died after resuscitation attempt; “You work to save a life and failed”

Feelings of worthlessness and abandonment; “I felt….lost,.…overlooked,….useless.”

## DISCUSSION

Teaching the essential communication skills of how to deliver difficult news of poor prognosis and death to family can be accomplished by including role-play communication exercises with simulated resuscitation scenarios. Role-play can be incorporated without a significant impact on session time and faculty resources. Major outcomes of our study included the following: 1) Most medical students reported limited real-life experiences with communicating difficult news; 2) The communication learning activities increased knowledge, comfort and confidence with communicating news of death and poor prognosis. When we explored student written self-reflections with intent to inform future curricula we found as a secondary outcome that resuscitations (both real and/or simulated) generated a variety of strong emotional responses in student learners.

Communication skills are taught in the pre-clerkship years to prepare students for clinical encounters in all undergraduate medical school curricula. However, these skills may decline by graduation if not reinforced.^27^,^28^ Real-life opportunities to deliver difficult news will vary from student to student, regardless of institution. Therefore, it is important for educators to create and provide opportunities with simulated scenarios so students can practice delivering news of death and poor prognosis, for example simulated resuscitations.^8^,^18^,^20^–^22^,^29^–^32^ Post-module, the levels of knowledge, comfort and confidence with delivering news of death and poor prognosis improved. Knowledge levels increased the most as compared to comfort and confidence.^8^,^33^–^35^ This may be due to the fact that unlike a gain in knowledge, comfort, and confidence in skills may take longer to build and require repeated practice over time. For some students the role-play exercise may actually serve to highlight the fact that these discussions may be much more difficult and complex than previously perceived.

The secondary aims of our project were to 1) encourage students to use written reflective inquiry for communication skills development and 2) explore these written learner experiences with the goal to inform future case scenario and curriculum design. Self-reflection exercises have also been shown to foster development of reflective capacity and enhance life-long learning and professionalism when used appropriately.^36^–^42^ Though we asked students to write on difficult communication, many however chose to express concern and lack of comfort with resuscitation experiences (both real and simulated). Therefore, the themes analysis has additionally provided valuable information about learner experiences and emotions during real and simulated resuscitations that may have implications for the school curriculum as well as the ED learning environment. This information may assist educators designing similar educational modules. In our institution, written self-reflection exercises occur in the early pre-clerkship years. However, based on student report it is clear that the current curriculum does not adequately reinforce the practice of written self-reflection, especially in the clerkship years. Students were initially overwhelmingly positive about the value of written self-reflections. However, the perceived value of written self-reflections declined after completing the exercise. We speculate that students may have 1) viewed this exercise as ‘added’ busy work for a clinical clerkship, 2) realized that writing about personal feelings may be more difficult than initially perceived, and 3) lacked validation of expressed emotions. Our study, as well as work by Dyrbye et al., also suggests that e-mail could be a useful modality to consider when offering formative feedback on clinical events/experiences that went well or did not go well and for self-reflective exercises.^28^,^43^

Patient resuscitations (both real and simulated) generated an emotional response in the learner. Frequently expressed learner emotions included anxiety, grief, sense of loss, and sense of failure. The emotions of professional failure and other personal reactions related to death and dying have been previously described.^9^,^11^,^18^,^30^,^31^,^33^,^44^–^49^ Many students also expressed ambivalence regarding ‘moving on to the next patient’ after a patient death. Some were unsettled by this routine ED practice whereas others felt it helped them cope with recent patient death. Clinicians also report that patient resuscitations are particularly stressful, especially those that end with patient death.^11^ The ED setting may present additional challenges to effective communication around resuscitation that include chaotic setting, time constraints, and no prior doctor-patient relationship.^8^,^45^ Stressful resuscitations may sometimes lead to unprofessional communications among healthcare workers and this was cited by many of our students under “things that did not go well.” In addition, in the ED setting students often reported feelings of worthlessness and abandonment while they acknowledged the well-organized efforts of the ED team in general. A suggestion to provide a sense of value as well as actively engage students in real resuscitations may therefore be to assign them simple tasks such as chest compressions.

In summary, we propose that communication modules should ideally routinely supplement ACLS and ATLS simulation resuscitation scenarios. We feel that this is an opportune time to closely integrate and reinforce communication skills with family members and encourage trainee self-reflection.^5^,^18^,^36^,^50^ Based on our learner self-reflection responses, we suggest that educators also explore ways to 1) address and validate the emotions felt by students and 2) address any negative role-modeling/communication that may occur during real and/or simulated resuscitation events.

## LIMITATIONS

Limitations of our study include the use of survey tools that were not previously validated, but internally developed by experts in EM and palliative care. Though the project was conducted at a single institution, the educational module is feasible to implement and adaptable across other settings. We target learners in a mandatory clerkship and results may differ with students on electives who may have a special interest in resuscitation or communication skills. We only evaluate pre-clerkship and post-clerkship self-report data (four weeks later). This self-report methodology is dependent on student perception and does not allow for objective measurement of knowledge and skills acquisition. Since the graduating senior students were not followed as they went on to the various residencies (with varied emphasis on communication skills training by discipline) we are unable to provide long-term data. As stated above, we did not provide one-on-one feedback to the learners on their written self-reflections and this may have led to the decline in scores related to the perceived value of the written exercise. This specific self-reflection format therefore may not have been constructed to provide maximal benefit to students.

## CONCLUSION

Simulated resuscitation case-based scenarios present an opportunity to closely integrate teaching of communication and self-reflection skills. A communication module with role-play increased trainee knowledge, comfort, and competence regarding communication of difficult news of poor prognosis and death after resuscitation. Educators may need to seek ways to address the strong emotions generated in learners with real and simulated patient resuscitations.

## Figures and Tables

**Table 1:** Emergency medicine pre-clerkship survey responses.

Questions	n=120 (%)
Self-reflection	12 (10)
Have you received specific training on self-refleciton?	
Have you ever used the following to discuss what went well in a patient encounter?	
Blog	21 (18)
Social	38 (33)
Diary	37 (32)
Email	76 (64)
Have you ever used the following to discuss things that did *not* go well?	
Blog	16 (64)
Social	27 (23)
Diary	34 (29)
Email	73 (61)
Communication training	
Specific training for closed-loop?	17 (15)
Is self-reflection a valuable tool for?	
Personal growth	108 (91)
Improvement in clinical practice	90 (75)
Lifelong learning	95 (80)
CDN of death	
Witnessed	96 (80)
Performed self	20 (17)
Role-played	104 (87)
CDN of poor prognosis	
Witnessed	109 (92)
Performed self	34 (29)
Role-played	110 (92)

*CDN*, communicating difficult news

**Table 2:** Comparison of pre and post emergency clerkship survey responses.

Questions	Pre	Post	p-value^*^
Is self reflection a valuable tool for?^1^			
Personal growth	90.8%	73.9%	<0.001
Improvement in clinical practice	80.5%	65.3%	0.005
Lifelong learning	80.5%	67.8%	0.011
Comfort with CDN^2^			
Death	Median 3.0	Median 3.0	0.006
	Mean 2.5	Mean 2.7	0.010
Poor prognosis	Median 3.0	Median 3.0	0.003
	Mean 2.7	Mean 2.9	0.005
Confident with CDN			
Death	Median 3.0	Median 3.0	<0.001
	Mean 2.6	Mean 2.8	<0.001
Poor prognosis	Median 3.0	Median 3.0	0.002
	Mean 2.7	Mean 2.9	0.001
Knowledge of CDN			
Death	Median 3.0	Median 3.0	<0.001
	Mean 2.8	Mean 3.2	<0.001
Poor prognosis	Median 3.0	Median 3.0	<0.001
	Mean 2.9	Mean 3.2	<0.001

*CDN*, communicating difficult news

1 p-values obtained via McNemar Chi-Square tests.

2 p-values for comparisons of medians obtained via Wilcoxon signed ranks tests; p-values for comparisons of means obtained via paired-samples t-tests.
